# About Zika Virus (ZIKV). Reply to Weidmann et al. Zika Virus Pathogenicity Versus Transmissibility. Comment on “Roozitalab et al. Distinct Virologic Properties of African and Epidemic Zika Virus Strains: The Role of the Envelope Protein in Viral Entry, Immune Activation, and Neuropathogenesis. *Pathogens* 2025, *14*, 716”

**DOI:** 10.3390/pathogens15010068

**Published:** 2026-01-09

**Authors:** Ashkan Roozitalab, Chenyu Zhang, Jiantao Zhang, Ge Li, Chengyu Yang, Wangheng Hou, Qiyi Tang, Richard Y. Zhao

**Affiliations:** 1 Department of Pathology, University of Maryland School of Medicine, Baltimore, MD 21201, USA; 2 State Key Laboratory of Molecular Vaccinology and Molecular Diagnostics, National Institute of Diagnostics and Vaccine Development in Infectious Diseases, School of Life Sciences, Xiamen University, Xiamen 361102, China; 3 Department of Microbiology, Howard University College of Medicine, Washington, DC 20059, USA; 4 Department of Microbiology and Immunology, University of Maryland School of Medicine, Baltimore, MD 21201, USA; 5 Institute of Human Virology, University of Maryland School of Medicine, Baltimore, MD 21201, USA; 6 Institute of Global Health, University of Maryland School of Medicine, Baltimore, MD 21201, USA

## 1. African vs. Asian Lineage: Pathogenicity and Host Adaptation

The authors emphasize on the long-standing codon adaptation of African ZIKV lineages to primate hosts, as this evolutionary framework helps explain several characteristics of African strains. Our observations complement this perspective by suggesting that codon adaptation alone does not fully account for the distinct behaviors we observed in human neural cells. In our study, the African strain MR766 exhibited stronger initial binding and higher replication in SH-SY5Y neural cells, while BR15 induced a more prominent innate immune response. These patterns support the idea that African lineage viruses are highly competent in neural cell infection, whereas the resulting cellular outcomes may depend more on host responses and tissue-specific factors than on replication efficiency alone.

## 2. Role of the E Protein in Viral Entry and Neuropathogenesis

We appreciate the commentary’s recognition of our findings that link early viral entry and replication dynamics to the envelope (E) protein [1,2]. Our chimeric virus analyses were designed to isolate these effects, and the results suggest that the E protein plays a central role in regulating viral attachment and replication kinetics, while associated structural components such as prM contribute to downstream permissiveness and cytopathic outcomes [1,3]. Although MR766 lacks the N154 glycosylation motif, our experiments indicate that its E protein still mediates efficient neural cell binding. This observation suggests that multiple structural features, including regions within domains II and III, may contribute to strain-specific interactions with neural cells.

## 3. Transmissibility vs. Pathogenicity in ZIKV Lineages

We agree with the authors that viral transmissibility and pathogenicity are shaped by distinct evolutionary pressures and do not necessarily evolve in parallel. As emphasized in the commentary, ecological, vector-related, and population-level factors play central roles in epidemic spread. Although our study did not directly examine transmissibility, available evidence suggests that the enhanced spread of Asian-lineage ZIKV strains, or epidemic success is driven primarily by improved fitness in Aedes mosquito vectors, transmission dynamics, viral persistence, and modulation of host immune responses, rather than by intrinsic replication efficiency or human codon usage alone. Consistent with these observations, our data show that the epidemic BR15 strain induces stronger innate immune activation in human neural cells than the African MR766 strain, which may limit cytopathic effects while allowing sustained viral presence. Together, these findings support the view that transmission efficiency and viral persistence, rather than cellular cytopathogenicity alone, were key contributors to the widespread human impact of Asian-lineage ZIKV strains.

## 4. Relevance of MR766 as a Representative African Strain

We acknowledge the commentary’s point that MR766 may not fully represent the genetic and phenotypic diversity of African ZIKV strains. At the same time, MR766 remains among the most extensively characterized African isolates, with well-documented neurotropism in experimental systems. This makes it a useful reference strain for mechanistic studies such as ours. We agree that the use of well-characterized reference isolates including a broader range of African isolates in future work would further enrich comparative analyses.

## 5. Interpreting Neurovirulence in the Context of Human Outbreaks

The commentary raises an important distinction between neurovirulence observed in experimental models and the clinical patterns documented during outbreaks. Our findings align with this distinction. While MR766 displayed strong neurotropic and cytopathic properties in vitro, BR15 elicited stronger antiviral and immunomodulatory responses in human neural cells.

While the stronger innate immune activation induced by the epidemic BR15 strain may mitigate direct cytopathic effects and preserve neurosphere integrity despite lower initial replication efficiency, excessive or dysregulated immune responses can also contribute to immune-mediated neuropathology, as has been observed in many encephalitic viral infections. Taken together, our results suggest that host immune activation is a critical modulator of neuropathogenic outcomes and that clinical manifestations likely arise from a complex interplay among viral genetics, transmission dynamics, and host immune responses [4]. This interpretation aligns with the authors’ conclusion that epidemic success is unlikely to be driven solely by intrinsic cellular pathogenicity.

## 6. Conclusions

We appreciate the contextual insights provided by Weidmann et al. [5] and regard their commentary as a valuable complement to our mechanistic findings. Taken together, both perspectives highlight the importance of integrating virological, immunological, and evolutionary considerations in understanding ZIKV biology. Our work suggests that the E protein is a major determinant of neural infection, while lineage-specific immune activation patterns influence downstream neuropathogenic outcomes. We hope that our study contributes useful mechanistic context to ongoing efforts aimed at understanding the diverse behaviors of ZIKV lineages.

## 7. Acknowledgment

We thank Weidmann et al. for their thoughtful commentary [5] on our recent publication, “Distinct Virologic Properties of African and Epidemic Zika Virus Strains: The Role of the Envelope Protein in Viral Entry, Immune Activation, and Neuropathogenesis”, by Roozitalab et al., *Pathogens* 2025 [3]. Their remarks provide a valuable perspective on ZIKV evolution, transmissibility, and the interaction between viral genetic determinants and host biology. We appreciate the opportunity to offer clarifications and contextualize our findings alongside the points they raise.
